# Improvement Happens: Impacting Health at its Roots: An Interview with Rishi Manchanda

**DOI:** 10.1007/s11606-014-2902-1

**Published:** 2014-06-04

**Authors:** Michael Hochman

**Affiliations:** AltaMed Health Services, 2040 Camfield Ave., Los Angeles, CA 90040 USA

R*ishi Manchanda, MD, MPH, is an Internist and Pediatrician who co-founded HealthBegins* (http://www.healthbegins.org/), *a network for health care professionals interested in addressing the social determinants of health within their health system. HealthBegins also provides consulting services and support to health systems that seek to integrate public health approaches with traditional medical services. Prior to founding HealthBegins, Dr. Manchanda was a clinician and the director of Social Medicine for a community health center in South Central Los Angeles. Dr. Manchanda recently released a TED Book titled "The Upstream Doctors", which describes his ideas for developing a clinician workforce with the tools to improve health at its roots. In this edition of Improvement Happens, JGIM Contributor Michael Hochman, MD, MPH talks with Dr. Manchanda about his vision for integrating an "upstream" approach within the health care delivery system.*

**JGIM:** How did you become interested in addressing the social determinants of health?

**Dr. Rishi Manchanda:** Prior to founding HealthBegins, I worked for several years in South Central Los Angeles as a clinician and a leader in the clinical system. While working there, I met a young woman named Veronica. Veronica had recurrent headaches that were accompanied by fatigue and malaise. These symptoms were interfering with her activities of daily living. She had sought care for her symptoms in the emergency room, where she had been worked up on multiple occasions with CAT scans, blood tests, and a lumbar puncture. These tests did not reveal the cause of her symptoms and she was given medication for pain and told to return if she didn’t get better.

But her headaches persisted. She began taking more sick days and she felt like she wasn’t doing enough for her young children. That was when Veronica first presented to my clinic.

When Veronica showed up, our Medical Assistant, using a screening tool to identify housing risk factors, discovered that she had mold in her home, water leaks, and roaches. This information was presented to me, along with her vital signs and chief complaint. During my encounter with Veronica, I realized that she had chronic allergies and migraine headaches—both directly tied to her poor living conditions.

I treated Veronica with medications for her symptoms, but more importantly, referred her to a community partner specializing in housing and health education. This organization sent a community health worker to her apartment. When Veronica came back three months later, her housing conditions were improved, and not coincidentally, she was feeling much better.

While working in South Los Angeles, I encountered situations like this virtually every day—and sometimes multiple times per day. I quickly realized the traditional skills I learned in medical school weren’t going to address many of these problems at their root, and I realized I would need a different approach. I looked to community stakeholders to identify the social and environmental conditions that I knew affected my patients’ health, and I began training my team to screen patients for these problems. I also began compiling community resources such as housing support available to them.

I also realized we were not the only clinic facing these challenges. In fact, there were a great number of people like us, but we were not yet connected. I realized I needed to do something to help other clinicians and managers address the social determinants.

**JGIM:** What problem are you trying to address with HealthBegins?

**RM:** We’re trying to address the problems at the intersection of health care and the social determinants of health. In plain English, we’re trying to improve the quality of care in medicine by helping health care practitioners better understand and address health where it begins—where we live, where we work, where we eat and play.

In the U.S. health care system, a lot of patients with complex medical problems also have complex and unmet social needs and problems in the environment or the larger community where they live. These patients are entering the health care system and seeking relief downstream for what are fundamentally upstream problems. In essence, we’re trying to treat disease while not addressing the social and environmental conditions that cause sickness.

At HealthBegins, we’re trying to make sure that health care systems have the knowledge and equipment to take care of those patients by impacting upstream events. So, if a patient comes in for a physical symptom but ends up disclosing—or the provider finds out—that they actually have some complex social problems at home or at work, then the team is quickly able to find a resource to help that patient.

**JGIM:** In your new Ted book “Upstream Doctors,” you refer to health care professionals who try to impact social determinants of health as “Upstreamists.” Can you define what an upstreamist is?

**RM:** An upstreamist refers to health care professionals who are equipped to transform their health care system and the social and environmental conditions that make people sick. An upstreamist should have an interest and passion for social justice. But an upstreamist is more than that. An upstreamist is someone who is charged with the responsibility in their own health care system to make sure that the practice of medicine—the routine practice of seeing patients, diagnosing them, treating them, referring them for care—includes the routine assessment and treatment of social determinants of health. The upstreamist has a specific job, not just a passion.

**JGIM:** Where does the term “upstreamist” come from?

**RM:** The upstreamist term comes from a well-known public health parable. In the way I tell the parable, three friends approach a river and hear the sounds of a child crying out for help. The three friends jump in and save the child. But to their dismay, they see more and more children and families and elderly coming down the stream in need of rescue. Again, they jump in to try to help. But soon, two of the friends realize that their third friend is no longer with them but instead is headed upstream. When his friends ask where he is going, he replies: “I’m going to find out who or what is throwing these people into the water.”

**JGIM:** Who in our health system can be an upstreamist? Is it a doctor, a public health expert, a community worker or all of the above?

**RM:** An upstreamist is a practitioner on the front lines of medicine who is working within their clinic or health system to figure out who or what is throwing patients into the water. Many upstreamists happen to be physicians, but there are nurses and other health professionals as well. The distinguishing feature is that they are on the front lines, working in the health care system while simultaneously thinking from an upstream perspective.

It is also important to emphasize that upstreamists need a specialized skill set to be effective, much like other health care specialists such as cardiologists. They often have public health training and a unique blend of leadership skills. They need to learn on the fly and be able to bring about change in an organization. The challenge for us now is how to create more upstreamists within health care.

**JGIM:** How is an upstreamist different from a public health professional?

**RM:** There is some overlap—and we do work closely with public health professionals—but the key distinction is that an upstreamist lives and works within health care on the front lines, typically as a clinician, facing the downstream consequences on a daily basis.

For too long, we’ve come to expect that health care professionals are simply supposed to manage the downstream consequences of upstream problems—as my friend Dr. Joe Greer, a physician leader who founded a medical clinic for the homeless in Miami, puts it. But that leaves health care professionals out of the mix. With the right support and training, some health care professionals can help identify and address upstream problems. They can transform their health care systems. And they can inform and leverage the vital efforts of public health professionals who work further upstream at a patient and population level.

I also want to make clear that we’re not talking about a system that transforms doctors into social workers. Rather, we’re looking to create a system in which health care providers can work at the top of their license with the presence of trained individuals adept at aligning care delivery with upstream work so that they collectively can better care for patients in a holistic way.

**JGIM:** Has this approach of helping those on the front lines look upstream been tried before?

**RM:** There’s a long history of attempts to integrate public health and clinical medicine. There have been notable successes in some communities across the U.S. and in fact, around the world. What is interesting is that it has not yet become part of the mainstream.

**JGIM:** What are some of the consequences when health care workers on the front lines aren’t equipped to address upstream problems?

**RM:** To name just a few, there are misdiagnoses or delayed diagnoses, high levels of inappropriate ER or hospital utilization, patient dissatisfaction from experiencing a health care system that isn’t asking questions that are most relevant to the context of their lives. In addition, you get provider dissatisfaction and challenges with provider recruitment and retention in underserved communities because they, themselves, lack the knowledge and the tools and the support to address these unmet social needs.

**JGIM:** In your book, you mention that socioeconomic circumstances can impact a person’s DNA—a phenomenon described by the developing field of epigenetics. Can you speak to this?

**RM:** The field of epigenetics looks at the ways in which environmental exposures can lead to differences in the way our genetic codes are expressed through physiological mechanisms. Genes can turn on or off in response to exposures in the environment. But we don’t have to wait for an epigenetic confirmation of what our experience already demonstrates: the social environment affects patients’ lives every day at an individual level and at a community level. Epigenetics is an interesting field worthy of further exploration, but it’s important for us not to lose sight of what we need to do now—which is to transform care and address the social environment of our patients.

**JGIM:** These are big challenges to take on. How can an upstreamist approach—and the work you do through HealthBegins—have an impact?

**RM:** At HealthBegins, we’ve become experts at designing and implementing innovations that tackle the complex problems in health care using public health methodology as well as LEAN startup principles (see Table 1) and system redesign and quality improvement techniques (i.e., systematic processes for making improvements that drive value). We work with health systems to test and spread these innovations.

**Table 1:** Lean Start-Uup Methodology

• A philosophy, related to “Lean Management,” calling for the elimination of waste in the start-up process, enabling ideas to be developed without large amounts of upfront resource investment
• Specifically, this means testing an idea or innovation on a small scale to obtain feedback, and using this feedback to continuously refine the idea or innovation
• Lean start-up methodology was popularized by the entrepreneur Eric Ries in a best-selling book: ***The Lean Startup: How Today***’***s Entrepreneurs Use Continuous Innovation to Create Radically Successful Businesses***

The second component of what we do is helping people learn about what works and what doesn’t with respect to impacting social determinants of health. We do this by providing training, mentorship, and support through workshops and soon an online learning platform. We’ve gone to different parts of the country to do this, from Boston, to here in Los Angeles, up to the Bay Area, and have been fortunate to work with partners such as the California Primary Care Association, the Prevention Institute and a number of others.

The last component involves building a network of like-minded innovators and experts interested in impacting the social determinants of health. Right now, we’re close to 700 members and we haven’t put any money into marketing or recruiting people. It’s purely just been a word-of-mouth enterprise.

**JGIM:** Can you give some specific examples of the projects and innovations that HealthBegins designs and implements?

**RM:** Sure, one example that we’re pretty proud of is based on a model we developed called Community Health Detailing. The Community Health Detailing model comes from the pharmaceutical detailing world, and we adapted it much like the academic detailing and public health detailing approaches have done before. First, we take a group of community stakeholders, and walk them through a modular curriculum to learn about social determinants in a very short, intensive course. Each module focuses on different social determinants like housing and food security and it’s very participatory. At the end of each module, there is a resource mapping exercise where a group of community stakeholders learns about resources in their own communities that address social determinants.

Our first pilot this past year involved 100 high school students in South Central Los Angeles who went through the program in three different cohorts. Collectively, these students mapped over 500 resources—from housing resources to food security resources—and in a way that’s very rigorous. They don’t just go online, but they call or even visit resources to vet them, and understand truly what kind of services they offer and who they serve. Some examples include affordable transportation services, reputable immigration assistance, and of course healthy food options, housing, recreation spots, and legal services.

We then take the resources mapped by the stakeholders and plug them into a resource mapping database that’s searchable by health care providers. It’s similar to a Yelp interface, so that a clinician or a care manager in a health care system can easily find resources for patients at the point of service when an unmet social need pops up Community residents—students in this case—then visit the clinicians to educate them about the connection between local social factors and their patients’ health. Our hope is that this model of engagement solves a real clinic problem by improving provider self-efficacy to help patients with unmet social needs.

**JGIM:** Is directing a patient to community resources sufficient? Aren’t you worried patients will fall through the cracks?

**RM:** This is a good point. We’re certainly mindful that just connecting patients to social service providers, while necessary, is not sufficient. Our students always remind us of this. One of the important next steps for us at HealthBegins is to work with technology partners to close the referral loop. For example, we’re exploring text messaging reminders to patients, as well as other strategies.

I would also point out that the resource database includes civic engagement and community organizing resources that can connect patients to larger collective efforts to address upstream problems.

**JGIM:** Do you have a sense yet whether health care providers are benefitting from the resources you mapped out? Are they using this ‘Yelp-like’ interface?

**RM:** The first clinic to test the resource is a UCLA clinic and they’re piloting it right now. We are seeing a lot of enthusiasm among the staff, especially the care managers who are excited about using an online database rather than having to use binders with tattered pieces of paper or information that can get outdated. Providers can make real-time online edits to resources on the database, including adjusting the name of relevant contacts, hours of operation, or changes in eligibility requirements.

**JGIM:** Did any surprising lessons come from the Community Detailing Project?

**RM:** One story I like to tell involves a student who entered a uniform supply company that makes and sells uniforms into the database. When we saw this resource pop up, I called up our team members because I was concerned. I didn’t expect this resource to come up.

I found out that the student had learned during our training modules about the role of employment and its impact on health. When we asked the student why he mapped the uniform company as a resource, he told us that if his father or a relative in his neighborhood needed to get a job in the local community, knowing where to find uniforms for local jobs could improve an applicants’ job prospects. The student had identified a resource that frankly we had not thought of to improve health through better employment. This story also illustrates how, as clinicians and educators, we need to be mindful of the context of our patients’ and students’ lives, and think broadly about how to impact social determinants like employment.

**JGIM:** So what’s next for the Community Health Detailing project?

**RM:** We just presented this project at the American Public Health Association in November 2013 in Boston and received a lot of wonderful feedback. We’re now looking at a multi-site intervention study to take the proof of concept pilot to the next level to look at measures of changes in provider efficacy, clinic productivity, and patient satisfaction and outcomes.

**JGIM:** I think a lot of people will be really supportive of the work you and your team are doing at HealthBegins. But as you noted before, the approach has not yet caught on or become part of the mainstream. How can you make this happen? Specifically, how will you make the business case for an upstreamist approach within our health system?

**RM:** We believe the key is to train and support health care professionals to achieve the triple aim in their health care systems by integrating upstream approaches in their work. If these approaches help to achieve the triple aim, the business case will follow.

The good news is that are already a lot of payers and providers who are thinking about the questions of social determinants. They may be doing so in different ways depending on the payer and the business model, but there’s already a lot of energy and excitement and some really concrete work that’s taking place.

In the capitated managed care environment, I think there’s a lot more movement towards trying to think about how to integrate the social determinants because there are incentives to optimize value. So, for instance, we’re already seeing a lot of people rallying around ways to improve care for the highest utilizers of the emergency room and hospital. Jeff Brenner’s work on high utilizers in Camden, NJ, popularized a few years ago in the New Yorker article by Atul Gawande, is a good example of an upstream approach in this regard.

I think the challenge is going to be for those providers that have a largely fee for service panel. The challenge for them is how to sustainably integrate social determinants with a fee for service system that does not support the necessary care team members or the work force changes that allow for upstreamists to work.

**JGIM**: But even in a managed care environment, as you think through the business case for an upstreamist approach, the payoff is often several years down the line. Are you concerned that even managed care organizations aren’t going to be willing to wait several years for a financial return?

**RM:** Now, we’re seeing a lot more movement around high utilizers because it’s something health care organizations can see the short-term potential return on investment.

However, I think that health care organizations will be more likely to take on social determinants interventions with a long tail and where the return on investment might take years when there is better integration of not just clinical providers but also community social service providers. If you start to think about more sophisticated accountable care organizations such as the one in Hennepin County in Minnesota or Vermont’s medical home structures or what’s happening in Oregon with their coordinated care model, we start to see some of the vanguard structures that incorporate not only clinical providers, but also social service providers. I think those kinds of structures and that level of integration will support investment in interventions that will take a longer period of time. This will allow for more interventions aimed at primary prevention or secondary prevention rather than just high utilizer work.

**JGIM**: How is HealthBegins funded?

**RM:** Right now, HealthBegins is self-funded and funded through contracts that we have with some of our partners where we provide training and consulting services. We haven’t gone for any grants yet, but we’re talking to some funders who have expressed some interest in supporting this work. We’re starting to evolve from the bootstraps startup phase to the next phase of development. It’s an exciting time.

**JGIM**: Do you think that ultimately you’ll be able to support HealthBegins with contracts with health systems or do you think in the long run your business model will be grant support?

**RM:** We see a mixed approach most likely. I think as we refine our services we will develop more contracts with health systems. We’ve also gotten a lot of interest in the mentorship and the workshops that we’ve been providing. I can envision us developing boot camps for upstreamists from different health care organizations. Through these boot camps, HealthBegins would play an incubator role in stimulating the upstreamist approach.

**JGIM**: Do you see any policy changes that could promote an upstreamist approach and interventions that impact social determinants of health?

**RM:** Absolutely. I would advocate for new payment methodologies to allow for reimbursements for the kinds of activities that really impact patients’ lives, specifically the care management work that happens between clinic visits. So much of what gets reimbursed for right now is the work that happens at a clinic visit, but we know that health really happens in the 5,000+ hours that are spent outside the health system walls every year.

I also think there’s an educational gap. There have been some healthy changes, for instance the reforms to the Medical College Admission Test that will go into effect next year with more questions about social and behavioral domains, but there’s still a lot more work to be done to provide the kind of ongoing education and learning collaboratives for frontline clinicians so that they can interact with upstream agents. And these activities could be supported with some policy changes.

**JGIM**: What are the implications of the work you are doing for Academic Medical Centers?

**RM:** Academic Medical Centers can play an integral role in promoting upstreamist work. AMCs can provide the health workforce with concrete skills to design and advance upstream interventions in clinics and health systems. At HealthBegins, we do a lot of work with AMCs in this regard. We find that health care professional students are often the most enthusiastic about the upstreamist approach.

Secondly, AMCs can provide technical assistance to local clinics and communities to support upstreamist work. AMCs have the intellectual resources to provide the data and creative ideas for designing and implementing upstreamist interventions.

**JGIM**: What would you say to others out there who want to become an upstreamist but don’t feel they have the resources to do it?

**RM:** I think it’s important for the upstreamists out there to start to see themselves as part of a community. As a first step, we need to create a taxonomy of interventions and approaches available to us because only then can we start to build a more integrated approach so that we all feel in it together.

